# Combining Information from Common Type 2 Diabetes Risk Polymorphisms Improves Disease Prediction

**DOI:** 10.1371/journal.pmed.0030374

**Published:** 2006-10-03

**Authors:** Michael N Weedon, Mark I McCarthy, Graham Hitman, Mark Walker, Christopher J Groves, Eleftheria Zeggini, N. William Rayner, Beverley Shields, Katharine R Owen, Andrew T Hattersley, Timothy M Frayling

**Affiliations:** 1 Department of Diabetes Research and Vascular Medicine, Peninsula Medical School, Exeter, United Kingdom; 2 Oxford Centre for Diabetes, Endocrinology, and Metabolism, Churchill Hospital, Headington, Oxford, United Kingdom; 3 Centre of Diabetes and Metabolic Medicine, Bart's and the London Queen Mary's School of Medicine and Dentistry, University of London, London, United Kingdom; 4 Department of Medicine, School of Medicine, Newcastle-upon-Tyne, United Kingdom; Lund University Hospital, Sweden

## Abstract

**Background:**

A limited number of studies have assessed the risk of common diseases when combining information from several predisposing polymorphisms. In most cases, individual polymorphisms only moderately increase risk (~20%), and they are thought to be unhelpful in assessing individuals' risk clinically. The value of analyzing multiple alleles simultaneously is not well studied. This is often because, for any given disease, very few common risk alleles have been confirmed.

**Methods and Findings:**

Three common variants (Lys23 of *KCNJ11,* Pro12 of *PPARG,* and the T allele at rs7903146 of *TCF7L2*) have been shown to predispose to type 2 diabetes mellitus across many large studies. Risk allele frequencies ranged from 0.30 to 0.88 in controls. To assess the combined effect of multiple susceptibility alleles, we genotyped these variants in a large case-control study (3,668 controls versus 2,409 cases). Individual allele odds ratios (ORs) ranged from 1.14 (95% confidence interval [CI], 1.05 to 1.23) to 1.48 (95% CI, 1.36 to 1.60). We found no evidence of gene-gene interaction, and the risks of multiple alleles were consistent with a multiplicative model. Each additional risk allele increased the odds of type 2 diabetes by 1.28 (95% CI, 1.21 to 1.35) times. Participants with all six risk alleles had an OR of 5.71 (95% CI, 1.15 to 28.3) compared to those with no risk alleles. The 8.1% of participants that were double-homozygous for the risk alleles at *TCF7L2* and Pro12Ala had an OR of 3.16 (95% CI, 2.22 to 4.50), compared to 4.3% with no *TCF7L2* risk alleles and either no or one Glu23Lys or Pro12Ala risk alleles.

**Conclusions:**

Combining information from several known common risk polymorphisms allows the identification of population subgroups with markedly differing risks of developing type 2 diabetes compared to those obtained using single polymorphisms. This approach may have a role in future preventative measures for common, polygenic diseases.

## Abstract 2

Combining information from several known common risk polymorphisms allows the identification of subgroups of the population with markedly differing risks of developing type 2 diabetes.

## Editors' Summary

**Background.:**

Diabetes is an important and increasingly common global health problem; the World Health Organization has estimated that about 170 million people currently have diabetes worldwide. One particular form, type 2 diabetes, develops when cells in the body become unable to respond to a hormone called insulin. Insulin is normally released by the pancreas and controls the ability of body cells to take in glucose (sugar). Therefore, when cells become insensitive to insulin as in people with type 2 diabetes, glucose levels in the body are not well controlled and may become dangerously high in the blood. These high levels can have long-term damaging effects on various organs in the body, particularly the eyes, nerves, heart, and kidneys. There are many different factors that affect whether someone is likely to develop type 2 diabetes. These factors can be broadly grouped into two categories: environmental and genetic. Environmental factors such as obesity, a diet high in sugar, and a sedentary lifestyle are all risk factors for developing type 2 diabetes in later life. Genetically, a number of variants in many different genes may affect the risk of developing the disease. Generally, these gene variants are common in human populations but each gene variant only mildly increases the risk that a person possessing it will get type 2 diabetes.

**Why Was This Study Done?:**

The investigators performing this study wanted to understand how different gene variants combine to affect an individual's risk of getting type 2 diabetes. That is, if a person carries many different variants, does their overall risk increase a lot or only a little?

**What Did the Researchers Do and Find?:**

First, the researchers surveyed the published reports to identify those gene variants for which there was strong evidence of an association with type 2 diabetes. They found mutations in three genes that had been shown reproducibly to be associated with type 2 diabetes in different studies: *PPARG* (whose product is involved in regulation of fat tissue), *KCNJ11* (whose product is involved in insulin production), and *TCF7L2* (whose product is thought to be involved in controlling sugar levels). Then, they compared two groups of white people in the UK: 2,409 people with type 2 diabetes (“cases”), and 3,668 people from the general population (“controls”). The researchers compared the two groups to see which individuals possessed which gene variants, and did statistical testing to work out to what extent having particular combinations of the gene variants affected an individual's chance of being a “case” versus a “control.” Their results showed that in the groups studied, having an ever-increasing number of gene variants increased the risk of developing diabetes. The risk that someone with none of the gene variants would develop type 2 diabetes was about 2%, while the chance for someone with all gene variants was about10%.

**What Do These Findings Mean?:**

These results show that the risk of developing type 2 diabetes is greater if an individual possesses all of the gene variants that were examined in this study. The analysis also suggests that using information on all three variants, rather than just one, is likely to be more accurate in predicting future risk. How this genetic information should be used alongside other well-known preventative measures such as altered lifestyle requires further study.

**Additional Information.:**

Please access these Web sites via the online version of this summary at http://dx.doi.org/10.1371/journal.pmed.0030374.

- NHS Direct patient information on diabetes
- National Diabetes Information Clearinghouse information on type 2 diabetes
- World Health Organization Diabetes Programme
- Centers for Disease ControlDiabetes Public Health Resource

## Introduction

An increasing number of common gene variants (minor allele frequency > 5%) reproducibly associate with polygenic disease. With some exceptions, such as complement factor H variation and age-related macular degeneration [1–3], these variants only mildly predispose to disease, with allelic ORs generally below 1.5. These common, low-penetrance risk alleles may explain much of the genetic component of complex multifactorial disease [4]. In addition to providing important etiological insights, identifying risk alleles could help define groups of people at relatively high or low risk of developing a disease. Some previous theoretical papers based on simulated data have demonstrated the potential utility of combining information from multiple common, low-penetrance variants for complex disease prediction [5,6]. However, studies using real data have been limited to either combinations of relatively rare variants [5], pairs of risk polymorphisms [7,8], variants that individually have not been established as risk alleles [9], and prospective cohorts that are disadvantaged by a small number of cases on follow-up [10].

Type 2 diabetes is a typical complex, polygenic disease for which several common risk alleles have been identified. In this study we have defined a reproducibly associated type 2 diabetes variant as one in which an association from a meta-analysis of published studies has reached genome-wide levels of significance. Using this definition, alleles of *PPARG* (Pro12) [11], *KCNJ11* (Lys23) [12–14], and *TCF7L2* (T at rs7903146) [15] have been reproducibly associated with type 2 diabetes. Individually, each of these polymorphisms only moderately predisposes to type 2 diabetes with odds ratios (ORs) ranging from ~1.15 for the Lys23 variant of the *KCNJ11* variant to ~1.50 for the rs7903146 variant of *TCF7L2*. Throughout this paper we use Pro12, Lys23, and “T at rs7903146” to refer to the risk alleles in the genes *PPARG, KCNJ11,* and *TCF7L2*, respectively.

The aim of this study was to examine the joint effects of replicated type 2 diabetes variants on disease risk in a large case-control study. To do this we studied 2,409 type 2 diabetes cases and 3,668 population-based controls, both cases and controls drawn from a population of white UK residents.

## Methods

### Participants

The clinical characteristics of the cases and controls are shown in Table 1. Informed consent was obtained from all participants. Participants have been described in detail [16]. Briefly, all participants with type 2 diabetes were unrelated and of white UK origin who had diabetes defined either by WHO criteria [17] or by being treated with medication for diabetes, and were recruited from four sources: (i) a collection of young-onset (defined as ≤ 45 years at age of diagnosis) patients with type 2 diabetes; (ii) probands from type 2 diabetic sibships from the Warren 2 sibling pairs described previously [18,19]; (iii) a collection of patients with type 2 diabetes (Warren 2 cases) diagnosed between ages 35 and 65 y, but not selected on family history; (iv) and probands from a collection of families that had either both parents available, or one parent and at least two siblings [20].

**Table 1:**
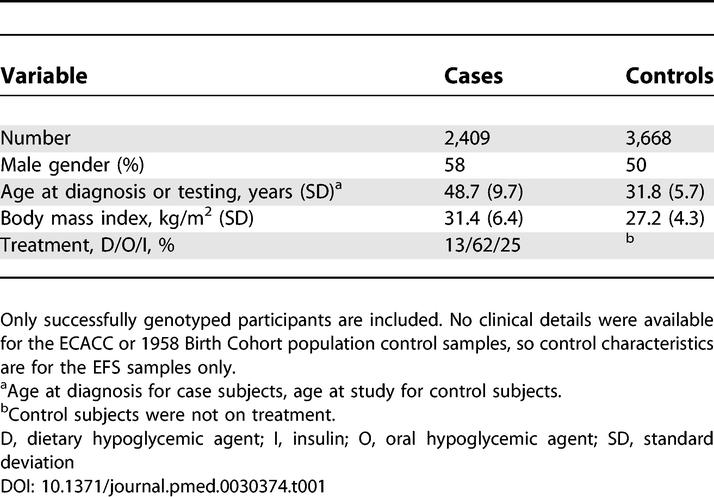
Clinical Details of Study Participants

Population control participants were all UK whites. These were recruited from three sources: (i) parents from a consecutive birth cohort (Exeter Family Study) with normal (<6.0 mmol/l) fasting glucose and/or normal HbA1_c_ levels (<6%; Diabetes Control and Complications trial corrected) [18]; (ii) a nationally recruited population control sample of UK whites obtained from the European Cell Culture Collection (ECACC), and (iii) an ongoing follow-up study of all people born in Great Britain during one week in 1958 (National Child Development Study, http://www.cls.ioe.ac.uk/Cohort/Ncds/mainncds.htm). Cases and families in which the proband had high GAD autoantibody levels (>99th percentile of the normal population) were excluded from the study. Known subtypes of diabetes (e.g., MODY, maturity onset diabetes of the young) were excluded by clinical criteria and/or genetic testing.

There was no evidence of heterogeneity of individual single nucleotide polymorphism (SNP) allele frequencies between case groups or control groups (all *p* > 0.01). We therefore pooled case groups and control groups in our analysis. The lactase polymorphism rs4988235, which varies 2-fold in frequency across the UK [21], is not associated with type 2 diabetes in our population (OR = 0.97 [95% CI, 0.90 to 1.06], *p* = 0.52), suggesting that population stratification is unlikely in our study (unpublished data).

### Genotyping

Except for the Pro12Ala variant in the 1958 birth cohort, genotyping was performed by KBioscience (Herts, UK) using a modified TaqMan-based assay, details of which can be found at their website (http://www.kbioscience.co.uk). The Pro12Ala genotypes in the 1958 birth cohort were genotyped using TaqMan. Genotyping accuracy, as determined from the genotype concordance between duplicate samples (9.5% of the overall sample) was 99.6%. The genotyping success rate was 94.7% for controls and 95.6% for cases. All polymorphisms were in Hardy-Weinberg equilibrium in cases and controls (all *p* > 0.05).

### Statistical Analysis

We used logistic regression for association tests. This method assumes a multiplicative allelic effect, such that each allele independently increases odds of disease. We tested for deviation from the logistic model by testing goodness of fit for both the individual SNP and the full model. For the interaction analysis we used case-control and case-only designs. The case-only analysis tests for independence of alleles in a case group, and can be more powerful than the case-control test, assuming that the variants are independent in the general population [22]. We used Quanto for power calculations [22]. To determine the positive predictive value of the type 2 diabetes risk alleles we used the likelihood ratio method of Yang et al. [5]. The likelihood ratios were estimated from the logistic regression model parameters. Using this approach the likelihood ratio is multiplied by the pretest probability of the disease to estimate the post-test probability of the disease. We used Stata SE version 9.1 (Stata, College Station, Texas, United States) to generate the receiver operating characteristic (ROC) curve and for calculating the area under the curve.

## Results

First we confirmed that the individual polymorphisms were predisposing to type 2 diabetes in these samples. The ORs for individual variants are shown in Table 2 and are similar to those observed in other large studies [11–13,15]. Some of the individual polymorphism results have been published previously [12,23], although for this study further case and control samples were tested to increase statistical power when looking for interaction between alleles. At each of these variants, our data (and that from the literature) are consistent with each risk allele independently increasing the odds of type 2 diabetes (i.e., individually all variants fit a multiplicative inheritance model); goodness of fit *p*-values are 0.81, 0.92, and 0.99 for Glu23Lys, Pro12Ala, and rs7903146, respectively.

**Table 2:**
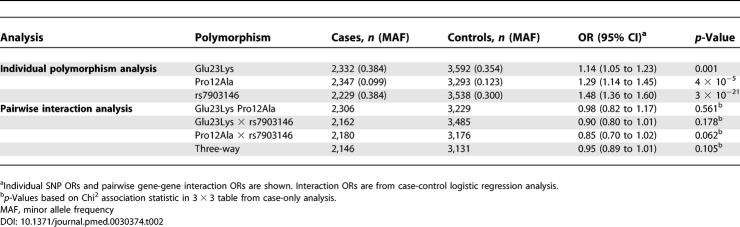
Individual SNP and Gene-Gene Interaction ORs

**Figure 1:**
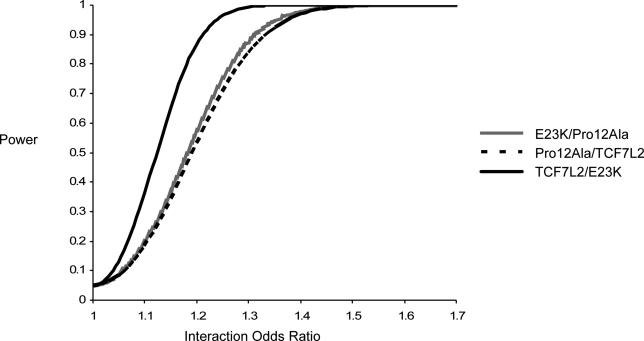
Power to Detect Interaction ORs for Combinations of SNPs The power calculations assume a multiplicative mode of inheritance. Estimates of allele frequencies and expected effect size were determined from the literature; our study frequencies and effects sizes are consistent with these. α was set at 0.05. The power calculations were performed using Quanto [22].

We next looked for potential gene-gene interaction between the three variants and type 2 diabetes status (i.e., deviation from a multiplicative model) using logistic regression. We used both case-only and case-control designs [22]. Power to detect interaction ORs for pairs of SNPs are shown in Figure 1. We had 80% power to detect pairwise interaction ORs of 1.18 to 1.28 at *p* ≥ 0.05, depending on the combination of polymorphisms. A pairwise interaction OR of 1.25 means, in the example of a pair of alleles each with main effect ORs of 1.2, that the joint OR is increased from 1.44 (no interaction) to 1.8 (i.e., 1.2 × 1.2 × 1.25).

Gene-gene interaction results are shown in Table 2. No pairwise or three-way combinations of genotypes showed evidence of interaction. While our results suggest that these variants increase risk in a simple multiplicative manner, it is important to note that we cannot rule out a range of other joint effects models. For example, our data are also consistent with an additive model of gene-gene interaction; however, because the ORs for the individual polymorphisms are low, our study is poorly powered to distinguish between an additive and multiplicative model.

**Figure 2:**
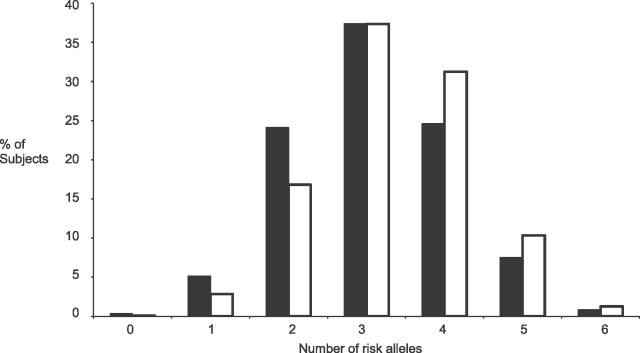
The Distribution of Risk Alleles in the Controls and Cases Controls are indicated by black bars, cases by white bars.

**Figure 3:**
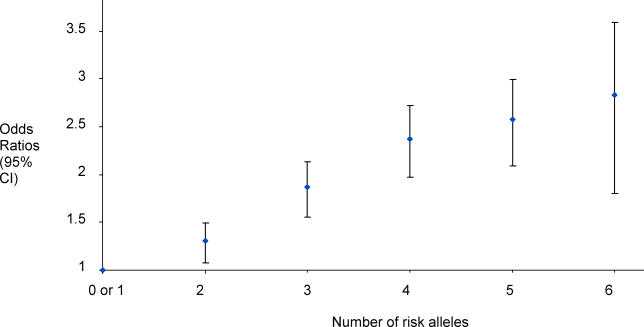
ORs and 95% CIs for Participants Carrying Increasing Numbers of Risk Alleles The reference group is individuals with zero or one risk allele.

The proportion of participants with increasing numbers of risk alleles in cases and in controls are shown in Figure 2. The ORs for type 2 diabetes for individuals carrying increasing numbers of risk alleles are shown in Figure 3, in comparison to the 4.5% of those that have no or only one risk allele. This reference group was chosen because only 0.25% of individuals carry zero risk alleles. The progressive increase in ORs is consistent with an independent effects multiplicative model (goodness of fit test *p* = 0.49). Each additional risk allele increased odds of disease by 1.28 (95% CI, 1.21 to 1.35) times. Participants with six risk alleles had an OR of 2.84 (95% CI, 2.08 to 3.87) compared to the reference group. Because of the relatively large influence of the rs7903146 on type 2 diabetes risk, Table S1 provides ORs for all different combinations of risk alleles. The 8.1% of participants who are double-homozygous for risk alleles at Pro12Ala and rs7903146 have an OR of 3.16 (95% CI, 2.22 to 4.50), *p* = 1.5 × 10^−10^, compared to the 4.3% in the reference group. If the 1% of persons with all six risk alleles are compared to the 0.25% of those with zero risk alleles, the OR is 5.71 (95% CI, 1.15 to 28.3), *p* = 0.03.

An alternative way to assess the impact of susceptibility alleles on disease risk is to use positive predictive values. Assuming a background risk of 5% in the general population, the probability of people with zero risk alleles developing type 2 diabetes is 2% compared to 10% for people with all six risk alleles.

## Discussion

This study examined how combining information from three highly replicated SNPs alters the relative risk of type 2 diabetes in case-control studies. It is the first study, to our knowledge, to look at the impact of the combined predictive value of three common (allele frequencies 0.3–0.88) genetic variants that have individually reached genomewide significance in meta-analysis. We showed that there is no evidence for interaction (within the limits of this study's power), and that the prediction of relative risk is greater when the three alleles are combined in a manner that is consistent with a multiplicative model.

**Figure 4:**
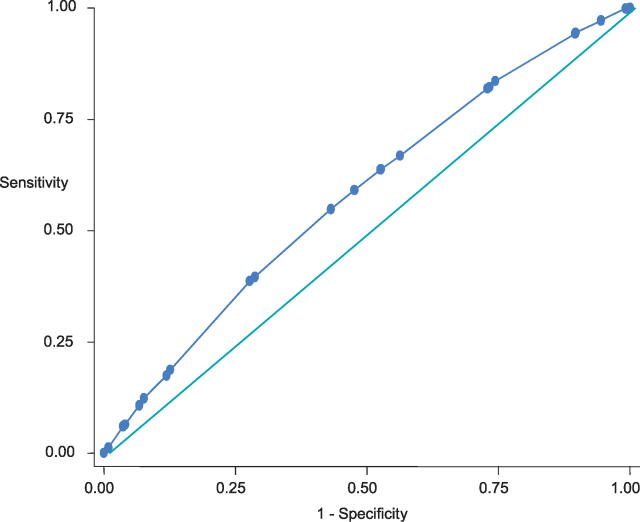
ROC for the Information Provided by the Glu23Lys, Pro12Ala, and rs7903146 Variants after Fitting a Logistic Regression Model AUC = 0.58.

Because there are only three confirmed common type 2 diabetes risk variants, the predictive power of this genetic information is still small compared to environmental and lifestyle influences such as body mass index and physical activity [24]. Considerable cause for optimism remains, however, that common type 2 diabetes risk polymorphisms will provide meaningful predictive information. Janssens et al. have suggested that the predictive power of genetic tests should be evaluated by the area under the ROC curve (AUC) [25]. The AUC is a measure of the discriminatory power of the test. A perfect test would have an AUC of 1; a test with no discriminatory power would have an AUC of 0.5. The ROC curve for the three polymorphisms described here is plotted in Figure 4; the area under the ROC curve is 0.58. There are two reasons to think that this figure will improve with time and that genetic screening in type 2 diabetes will eventually prove useful. First, the number of reproducibly associated variants is likely to increase rapidly with large clinical resources and the technology to perform whole-genome association studies in place. Yang et al. estimated that 20–25 risk variants with allele frequencies greater than 0.1 and ORs of 1.5 are required for an AUC of about 0.8 [5,26]*.* Second, the low cost of genotyping may mean identifying a small proportion of individuals at high risk would still be justifiable if preventative measures can be found.

One minor limitation of our study is that our cases are enriched for patients with a family history of type 2 diabetes and a young age of diagnosis. This may mean that, compared to the general type 2 diabetes population, we have overestimated the risk due to these variants. This should be offset, to some extent, by our use of a relatively young population-based cohort as our control group, approximately 5% of whom will develop diabetes later in life. We also note that the ORs we observed are consistent with those from other large studies in the literature. However, to assess the applicability of our findings to the general population, further studies will be needed—ideally large, prospective cohort studies. This will require use of future resources such as the UK Biobank, which is currently in the early stages of recruiting 500,000 individuals from the general UK population [27]. These very large-scale studies will also be needed to give us the adequate power to compare risk of disease in patients with the high numbers of risk alleles, and to identify subtle interaction effects.

It has been suggested that gene-gene and gene-environment interactions play a major role in complex disease predisposition. We found no evidence of gene-gene interaction between the variants we studied and type 2 diabetes risk. If some of the yet-unidentified variants interact with each other and/or with environmental factors, we will be able to further (and perhaps dramatically) increase the predictive power of polygenic variant information. However, based on our results, even in the absence of interaction the combination of information from several common, low-penetrance alleles may provide a good level of predictive power in persons with high numbers of risk alleles.

Despite limitations to this study, we feel that important conclusions can be drawn from our results. Our data support the idea that, while individual polygenic susceptibility variants may be of limited use in disease prediction, the combined information from a number of these variants allows the identification of groups of people at high and low risk of developing a complex disease [5,6]. This approach may allow the targeting of preventative measures to individuals at high risk of disease, although many further studies will be required to examine the efficacy of preventative measures in groups with high-risk genotypes.

In conclusion, although individual susceptibility alleles only moderately increase the risk of type 2 diabetes, the risk is multiplicatively increased when risk alleles are combined. The combined information from risk alleles allows the identification of subgroups of the population with odds for disease significantly greater than when using a single polymorphism.

## Supporting Information

Table S1 ORs and 95% CIs for Different Combinations of Risk Alleles Data are given for participants carrying differing combinations of risk alleles compared with those with zero *TCF7L2* risk alleles and zero or one Glu23Lys/Pro12Ala risk allele for type 2 diabetes status (Ref). NS, no participants
(41 KB DOC)

### Accession Numbers

The RefSeq (http://www.ncbi.nlm.nih.gov/RefSeq) accession numbers for *TCF7L2* is NM_030756; for *PPARG* is NM_005037; for *KCNJ11* is NM_000525 and for *Lactase* is NM_002299.

## Abbreviations

AUC: area under the receiver operating characteristic curve
CI: confidence interval
OR: odds ratio
ROC: receiver operating characteristic
SNP: single nucleotide polymorphism
