# Supplementary material for: Combining Information from Common Type 2 Diabetes Risk Polymorphisms Improves Disease Prediction
Source: PLoS Med. 2006 Oct 3;3(10):e374. doi: 10.1371/journal.pmed.0030374 (PMC1584415; doi:10.1371/journal.pmed.0030374)
Supplement: Supplementary file 1 [file pmed.0030374.st001.doc]

**Supplementary Table 1.** Odds ratios and 95% confidence intervals for subjects carrying differing combinations of risk alleles against subjects with zero *TCF7L2* risk alleles and zero or one E23K/Pro12Ala risk allele for type 2 diabetes status (Ref). NS = No subjects.

| **TCF7L2**  **(n risk alleles)** |  | | | **Pro12Ala**  **(n risk alleles)** | | |
| --- | --- | --- | --- | --- | --- | --- |
|  |  | | | **0** | **1** | **2** |
|  | **0** | **E23K** | **0** | Ref | Ref | 1.39 (1.00, 1.95) |
|  |  |  | **1** | Ref | 1.09 (0.72, 1.67) | 1.79 (1.30, 2.48) |
|  |  |  | **2** | 2.86 (0.39 20.79) | 2.44 (1.37, 4.35) | 1.80 (1.22, 2.66) |
|  |  |  |  |  |  |  |
|  | **1** | **E23K** | **0** | 3.82 (0.83, 17.57) | 1.63 (1.07, 2.50) | 2.07 (1.49, 2.88) |
|  |  |  | **1** | 0.95 (0.33, 2.74) | 1.86 (1.25, 2.77) | 2.59 (1.87, 3.58) |
|  |  |  | **2** | 2.86 (0.56, 14.58) | 1.88 (0.98, 3.59) | 2.34 (1.59, 3.45) |
|  |  |  |  |  |  |  |
|  | **2** | **E23K** | **0** | NS | 3.52 (1.86, 6.64) | 3.06 (2.02, 4.64) |
|  |  |  | **1** | 2.86 (0.39, 20.80) | 3.64 (2.01, 6.58) | 3.31 (2.20, 5.00) |
|  |  |  | **2** | NS | 2.29 (0.86, 6.08) | 2.97 (1.61, 5.50) |
